# Antinociceptive effects of intrathecal Neuropeptide B/W receptor 1 agonists in mouse acute nociception, peripheral neuropathy, and inflammatory pain models

**DOI:** 10.21203/rs.3.rs-6559172/v1

**Published:** 2025-05-16

**Authors:** Yuma T. Ortiz, Thuy Nguyen, Jenny L. Wilkerson

**Affiliations:** Texas Tech University Health Sciences Center; RTI International; Texas Tech University Health Sciences Center

**Keywords:** Neuropeptide B/W Receptor 1, Neuropeptide B, peptidomimetic, pain, neuropathic pain, hot plate latency, inflammatory pain

## Abstract

**Background:**

The Neuropeptide B/W Receptor 1 (NPBWR1) system, including its two endogenous ligands, Neuropeptides B and W (NPB and NPW), has garnered interest as potential target to develop novel analgesics. Behavioral studies were typically conducted with exogenously administered endogenous ligands. In this study, we examined truncated NPB-23 and its peptidomimetic RTIBW-16 in a panel of antinociceptive assays including the hot plate, carrageenan-induced inflammatory, and paclitaxel chemotherapy-induced peripheral neuropathy (CIPN) pain assays.

**Methods:**

Male and female C57BL/6 mice underwent testing in the hot plate acute nociception assay. After a minimum one-week washout, mice were enrolled in the carrageenan inflammatory pain model, receiving intraplanar carrageenan (0.3% carrageenan in a 20 μL). Separate mouse cohorts received a cycle of intraperitoneal paclitaxel injections (cumulative dose 32 mg/kg). The von Frey assay was utilized to assess CIPN and carrageenan-induced allodynia.

**Results:**

NPB-23 and RTIBW-16 dose-dependently produced thermal antinociception, attenuated CIPN allodynia and carrageenan-induced allodynia with some differences regarding onset time, potency and duration of action. In the hot plate assay, RTIBW-16 showed earlier onset but shorter duration of action than NPB-23 with similar maximum peak effects. Both compounds were statistically equipotent in the reversal of mechanical allodynia induced by either paclitaxel or carrageenan. RTIBW-16 maintained a longer duration of action than NPB-23 in CIPN assay.

**Conclusions:**

Both NPBWR1 agonists alleviated thermal and inflammatory pain. Notably, we demonstrated for the first time that NPBWR1 agonists exhibited analgesic effect in the CIPN model. Our findings highlight NPBWR1 as a promising target for developing analgesics with novel mechanisms.

## Introduction

Pathological pain encompasses chronic pain conditions such as neuropathic pain, inflammatory pain, and centralized pain syndromes [1–4]. Unlike acute pain, which serves as a protective mechanism, chronic pathological pain persists beyond normal healing and often arises from maladaptive changes in the nervous system [5–7]. It affects over 30% of the population worldwide, with a notable impact on quality of life, mental health, and daily functioning [8]. The global burden is profound, contributing to increased healthcare utilization, loss of productivity, and economic costs exceeding billions of dollars annually [8]. Pathological pain is intricately linked to conditions like diabetes, cancer, and autoimmune diseases, further amplifying its prevalence and impact [1, 9, 10]. Despite advances in pain management, opioids remain the only treatment for chronic moderate to severe pain. While effective, their use is restricted by significant limitations, including dependence, tolerance, and respiratory depression. The complexity of pain mechanisms and individual variability further challenge the development of effective treatments, emphasizing the urgent need for targeted research and innovation in therapeutic approaches [8].

The neuropeptides B and W (NPB and NPW) are endogenous ligands of the Neuropeptide B/W Receptors 1 and 2 (NPBWR1 and NPBWR2) [11]. Together, they have been implied to play a role in pain signaling pathways [12, 13]. NPBWR1 is expressed in brain and peripheral organs of both humans and rodents whereas NPBWR2 is not found in rodents [14]. Within the central nervous system (CNS), NPBWR1 expression was found in cerebellum, prefrontal cortex, hippocampus, amygdala, and spinal cord [12–16]. In the peripheral nervous system, NPBWR1 has been identified on both human and rat myelinating Schwann cells under basal conditions and was found to be overexpressed in neuropathic conditions [17]. A recent study utilizing immunohistochemistry demonstrated that NPBWR1 and μ opioid receptor are colocalized in the superficial dorsal horn of rat spinal cord [18].

In preclinical animal studies, mice lacking NPB developed hyperalgesia in both the formalin and acetic acid inflammatory pain models but responded normally in acute hot plate nociceptive pain and non-inflammatory pain models [19]. Exogenous intracerebroventricular (i.c.v.) administration of synthetic NPB produced thermal antinociception and decreased paw licking in rat carrageenan and formalin models, respectively [20]. Intrathecal (i.t.), but not i.c.v., administration of exogenous NPW and NPB reversed mechanical allodynia, or light touch sensitivity in the rat partial nerve ligation neuropathic pain model [21]. In another study that utilized rats intrathecal NPB and NPW attenuated formalin-induced nociceptive behaviors as well as carrageenan-induced mechanical allodynia but did not alter carrageenan-induced thermal hyperalgesia or acute hot plate nociception [22]. In a rat formalin test, i.c.v. injection of NPW into different brain regions revealed that its analgesic effects were mediated through the activation of the descending pain modulatory system [23]. Morphine co-administered with either NPB or NPW synergistically alleviated both inflammatory and neuropathic pain as well as significantly reduced morphine-induced conditioned place preference and constipation [18].

So far, behavioral studies have mostly been conducted in rats and relied on exogenous administration of endogenous ligands. NPB is a 29 amino acid peptide with a bromine at the C-6 position of the indole moiety in the N-terminal tryptophan (Trp) [11, 20, 24]. The truncated NPB-23 with the N-terminal 23 amino acid residues and no bromine at the N-terminal Trp was found to exhibit equipotent in vitro NPBWR1 agonist activity [24, 25]. NPW exists in two functional peptide forms consisting of 23 and 30 amino acid residues [24, 26]. NPB exhibits a higher affinity for NPBWR1, while NPW binds to both NPBWR1 and NPBWR2 with similar affinities [26]. Our group and others have studied the structure-activity relationship of NPB and identified that the pharmacophore for NPBWR1’s agonist activity consists of two epitopes, N-terminal WYK and C-terminal GRAAGLL [25, 27]. From this understanding, we developed RTIBW-16, a NPB peptidomimetic with a shorter sequence of 13 amino acid residues exhibited similar in vitro potencies while exhibiting improved metabolic stability in rat plasma [27].

In the current study we sought to evaluate the effects of i.t. administration of NPB-23, a truncated human NPB without a bromine at the N-terminal Trp residue and RTIBW-16 using a panel of mouse pain models including the hot plate assay for acute nociception, a chemotherapy-induced peripheral neuropathy (CIPN) and a carrageenan-induced inflammatory pain models. The hot plate assay is a widely used tool for screening novel analgesics [28–30]. CIPN is a clinically relevant that mimics the debilitating sensory and pain symptoms experienced by cancer patients undergoing chemotherapy, providing a valuable tool for developing therapies to alleviate this common side effect [1, 5, 10]. The carrageenan-induced pain model is a standard tool for evaluating potential analgesics for conditions like arthritis and other inflammatory disorders [20, 22, 31]. By profiling across various pain models, we aim to investigate the roles of NPBWR1 agonists in acute nociception, neuropathic and inflammatory pain.

## Materials and Methods

### Animals

All studies were conducted in compliance with the National Institutes of Health Guide for the Care and Use of Laboratory Animals and approved by the Texas Tech University Health Science Center Institutional Animal Care and Use Committee (IACUC number 21034). A total of 44 adult male and 44 adult female (22–29 g upon arrival) C57BL/6 (Jackson Laboratories, Bar Harbor, ME) mice were used in this study. Mice were split into cohorts (n = 8/group) and used for either the chemotherapy-induced peripheral neuropathy model or the hotplate latency assay with later use in the carrageenan paw inflammation model. Housing facilities for mice were maintained at a consistent temperature (20–22 °C)-, humidity (55% ± 10%)-, and light-schedule (12 hr light/dark; lights on at 0700) which were approved by the Association for Assessment and Accreditation of Laboratory Animal Care. Food and Water were available *ad libitum* for all mice.

### Drugs and Dosing

NPB-23 (Trp-Tyr-Lys-Pro-Ala-Ala-Gly-His-Ser-Ser-Tyr-Ser-Val-Gly-Arg-Ala-Ala-Gly-Leu-Leu-Ser-Gly-Leu-NH_2_) and RTIBW-16 ([desaminoTrp^1^]-Tyr-Lys-Ava-Ava-Ava-Gly-Arg-Ala-Ala-Gly-Leu-Leu-NH_2_, compound **30** in Ref. 27) were synthesized as previously reported [27]. RTIBW-16 and NPB-23 were administered via i.t. injections between L4 and L5 vertebrae with an injection volume of 5 μL. The dosing of these compounds was based on previous literature [21, 22, 27].

Paclitaxel was purchased from Bio-Techne (Minneapolis, MN) and was administered in a vehicle solution containing a 1:1:18 ratio of ethanol (100%), polyethylene glycol monooleyl ether (Tokyo Chemical Industry America; Portland, OR), and saline (0.9% w/v NaCl) as previously described [22, 24]. A dosing cycle of paclitaxel consisted of a total of four intraperitoneal (i.p.) paclitaxel injections (8 mg/kg per injection, given every other day [29, 32]. Carrageenan was purchased from Sigma-Aldrich (St. Louis, MO) and made into a 0.3% w/v solution in saline for injections [33].

### Intrathecal Injections

Intrathecal injections were conducted as previously described [34–36]; injections occurred between the L4 and L5 vertebrae. Briefly, an ‘injection catheter’ made from a 27-gauge needle and polyethylene (PE) 20 tubing was connected to a 10 μl Hamilton syringe. Injections occurred over 5 sec and were generally accompanied by a hind paw twitch response. A 100% motor recovery rate was observed.

### Acute Nociception Hot Plate Assay

Acute nociception measurements were collected via the hot plate latency assay as previously described [37]. Mice were placed on a heated (52 °C) enclosed Hot Plate Analgesia Meter (Columbus Instruments, Columbus, OH). The latency to jump/lick/shake/flick a hind paw was recorded. A 30 second cut-off was used to prevent tissue damage. Latencies were recorded prior to administration of compounds and at each time point over the 24-hour study (0.5, 1, 1.5, 2, 3, 4, 6, 24 hr time points). Latency measurements were taken by observers blinded to treatment conditions. Mice were given a 1-week washout period between test days.

### Paclitaxel CIPN Model

To assess mechanical allodynia within CIPN, mice were habituated to the von Frey testing environment for four consecutive days in 30-minute sessions prior to the first testing session. Baselines were measured as previously described using von Frey monofilaments (North Coast Medical; Morgan Hills, CA) to establish responses to light mechanical touch before paclitaxel or vehicle administration [24]. During testing mice were placed atop a wire mesh screen with spaces 0.5 mm apart. Mice were singly placed beneath an inverted wire mesh basket (8 cm diameter, 15 cm height) and allowed to habituate to the apparatus for 30 minutes before testing. The von Frey assay utilizes a series of calibrated monofilaments (0.4–4.0 g stimulus intensity), applied to the left and right plantar surface of the hind paws utilizing the “up-down” method [32]. Monofilament application was done in a manner avoiding sequential application to the same paw (i.e., left, right, left, right, as opposed to left, left, left, right). Lifting, licking, or shaking of the tested paw upon filament application was considered a response. Five responses out of five monofilament stimulations were coded as the minimum force required to elicit responses within the von Frey assay. Mice were tested with experimental compounds starting the day after finishing paclitaxel treatment and were given at least a 1-week washout period between test days. As mechanical allodynia in this model lasts through 3 months [32], animals experienced 5–7 test days. Reported measurements from the von Frey assay are mean response thresholds collected from both the left and right hind paw of each subject as paclitaxel produces bilateral allodynia of similar magnitude in both hind paws. Responses from the von Frey assay were measured over a 24-hour time course (1, 2, 3, 4, 6, 24 hr timepoints). Mechanical allodynia assessments were conducted by observers blinded to treatment conditions.

### Carrageenan Inflammatory Pain Model

After use in the hot plate latency assay, the same cohort was then used for the carrageen model of inflammatory pain. Mice were given at least a one-week washout period between experimental use. Mice were habituated to the von Frey apparatus and were assessed using the same “up-down” method as described earlier [38]. The carrageenan inflammatory pain model was induced as previously described [33]. Briefly, an intraplantar injection of 0.3% w/v carrageenan in a 20 μL volume using a 27-gauge needle was administered into the right hind paw. Carrageenan was injected into the right hind paw. The left paw served as an internal control for each subject. The change in paw thickness from baseline was assessed with digital caliper measurements. Paw thickness and von Frey responses were measured over a 24-hour time course (1, 2, 3, 4, 6, 24 hr timepoints), with paw thickness measurements occurring immediately after the von Frey assay at each time point. Mechanical allodynia assessments and paw thickness measurements were conducted by observers blinded to treatment conditions. Intraplantar administration of carrageenan and digital caliper measurement of paw edema were conducted with mice restrained utilizing a plexiglass mouse restrainer (Stoelting Co., Wood Dale, IL)

### Data analysis

Hot plate data were converted to percent maximum possible effect (% MPE) with the following equation: ([experimental test value – baseline value) / (30 sec – baseline value)] * 100). Behavioral data were analyzed using a repeated measures two-way analysis of variance (ANOVA) for RTIBW-16 and NPB-23 treatment time course analysis with time as one factor and compound dosing as a second factor [35]. Individual treatment effects were analyzed using repeated measures one-way ANOVA. Dunnett’s test was used for post hoc analysis following a significant ANOVA result. Two tailed, paired t-tests were conducted to compare the magnitude of baseline von Frey measurements to their respective pre-paclitaxel and pre-carrageenan measurements. A *p* value of < 0.05 was considered statistically significant. As no significant sex effect was observed, male and female data were collapsed. If the mean effect of treatment did not produce a 50% or greater effect, an effective dose (ED_50_) value was not generated. When the mean effect of a drug in an experiment was greater than 50%, the ED_50_ values and corresponding 95% confidence limits were calculated using linear regression, where slopes were allowed to vary [39]. The computer program GraphPad Prism version 10.3 (GraphPad Software Inc.; San Diego, CA) was used in the statistical analyses. Data are plotted as mean ± the standard error of the mean.

## Results

### Intrathecal RTIBW-16 and NPB-23 produced acute antinociception

**Figure 1:**
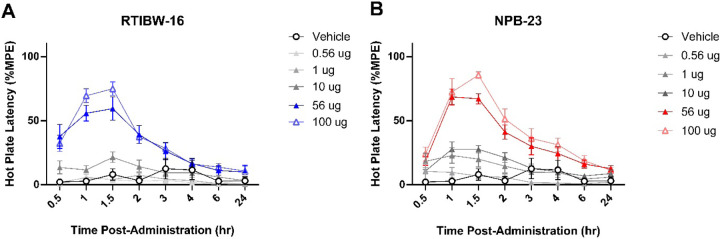
Intrathecal RTIBW-16 and NPB-23 produce dose- and time-dependent antinociception in the hot plate assay. RTIBW-16 (A) produced antinociception in the hot plate latency assay at the doses of 100 (blue, hollow triangle) and 56 μg (blue, filled triangle) through 2 hrs after administration. Similarly, NPB-23 (B) at doses of 100 (red, hollow triangle) and 56 μg (red, filled triangle) produced antinociception through 6 hrs after administration. Abscissae: time points in hours; ordinates: stimulus intensity to illicit paw withdrawal. Filled data points (black) indicate significance from vehicle measurements (*p* < 0.05). Data reflect mean ± SEM, n = 8.

Intrathecal vehicle administration did not alter response latencies (p = 0.268) in the hot plate assay. At 56 and 100 μg, both RTIBW-16 (Fig. 1A) and NPB-23 (Fig. 1B) produced significant antinociceptive effect as early as 0.5 hour and peaked at 1.5 hours after administration. At 100 μg (*F* (2.17, 15.18) = 11.73, *p* < 0.001) and 56 μg (*F* (2.08, 14.56) = 34.01, *p* < 0.001) of RTIBW-16 produced significant antinociceptive effects through 2 hours after administration. A two-way ANOVA confirmed a significant interaction between RTIBW-16 treatment and time (*F* (49, 343) = 7.68, *p* < 0.001). Similarly, 100 μg (F (1.44, 10.06) = 28.51, p < 0.001) and 56 μg (*F* (1.70, 11.87) = 44.08, *p* < 0.001) NPB-23 produced significant antinociception up to 6 hours following administration. A two-way ANOVA of NPB-23 confirmed a significant interaction between treatment and time (*F* (35, 245) = 9.61, *p* < 0.001). At 1 hour timepoint, the calculated ED_50_ values of RTIBW-16 and NPB-23 were 43.86 (25.09–73.21) and 18.36 (10.17–33.15) μg respectively (Table 1). Generally, in the hot plate assay, RTIBW-16 showed greater antinociceptive effect earlier at 0.5 hour timepoint but shorter duration of action than NPB-23. Both ligands produced similar maximum antinociceptive effects at their peak time of 1.5 hours.

### Intrathecal RTIBW-16 and NPB-23 Reversed Paclitaxel-Induced Mechanical Allodynia

**Figure 2:**
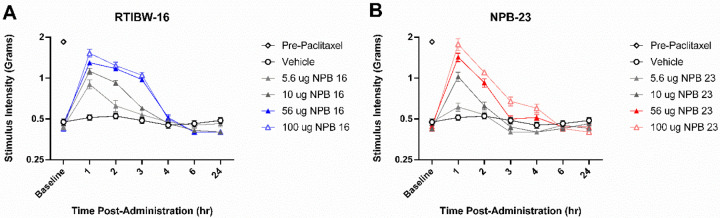
Intrathecal RTIBW-16 and NPB-23 produce dose- and time-dependent antinociception in the paclitaxel CIPN model. In the CIPN model RTIBW-16 (A) reversed mechanical allodynia for up to 3 hours following treatment with the 100 (blue, hollow triangle) and 56 μg (blue, filled triangle) doses. NPB-23 (B) produced antinociceptive effects at similar dosing (100 μg and 56 μg, red hollow and filled triangles, respectively) in the CIPN model up to 2 hrs following treatment. Abscissae: time points in hours; ordinates: stimulus intensity to illicit paw withdrawal. Filled data points (black) indicate significance from vehicle measurements (*p* < 0.05). Data reflect mean ± SEM, n = 8.

A separate cohort of mice were utilized to examine RTIBW-16 (Fig. 2A) and NPB-23 (Fig. 2B) in a model of paclitaxel-induced peripheral neuropathy. Following paclitaxel administration, mice displayed significant (*p* < 0.001) mechanical allodynia compared to pre-paclitaxel baselines. Vehicle treatment did not alter mechanical allodynia thresholds.

Both ligands produced maximum effects at 1 hour. RTIBW-16 significantly attenuated CIPN mechanical allodynia from 1 to 3 hours post administration. Specifically,100 μg (*F* (2.07, 31.1) = 80.8, *p* < 0.001), 56 μg (*F* (2.8, 42.04) = 209.1, *p* < 0.001), and 10 μg (*F* (2.38, 35.62) = 86.24, *p* < 0.001) produced dose-related mechanical allodynia reversal. A two-way ANOVA confirmed a significant interaction between RTIBW-16 treatment and time in the CIPN model (*F* (5.56, 83.43) = 28.5, *p* < 0.001). NPB-23 significantly attenuated mechanical allodynia for 2 hours following 100 μg (*F* (1.33, 19.89) = 54.57, *p* < 0.001) and 56 μg (*F* (1.97, 29.54) = 59.64, *p* < 0.001) administration. A two-way ANOVA confirmed a significant interaction between NPB-23 treatment and time in the CIPN model (*F* (3.94, 59.16) = 23.09, *p* < 0.001). At 1 hour timepoint, the calculated ED_50_ values of RTIBW-16 and NPB-23 were 57.79 (53.13–95.06) and 45.06 (33.91–59.88) μg respectively (Table 1). At 1 hour timepoint, NPB-23 was able to reverse the CIPN mechanical allodynia back to the baseline. RTIBW-16 maintained a longer duration of action than NPB-23. Both ligands were statistically equipotent in the reversal of mechanical allodynia.

### Intrathecal RTIBW-16 and NPB-23 Blocked Carrageenan-Induced Mechanical Allodynia and Edema

**Figure 3:**
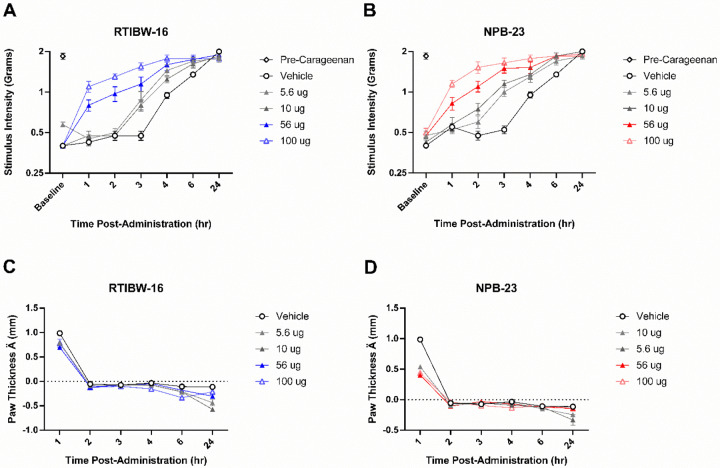
Intrathecal RTIBW-16 and NPB-23 decrease mechanical allodynia and inflammation in a model of carrageenan-induced inflammatory pain. RTIBW-16 (A) at doses of 100 μg (blue, hollow triangle) and 56 μg (blue, filled triangle) fully attenuated peak carrageenan-induced mechanical allodynia. NPB-23 (B) produced similar antinociceptive effects at similar dosing (100 μg and 56 μg, red hollow and filled triangles, respectively). RTIBW-16(C) and NPB-23 (D) treatment resulted in reduced initial swelling at the same time point of 1 hour. Abscissae: time points in hours; ordinates (A, B): stimulus intensity to illicit paw withdrawal; (C, D) change in paw thickness compared to baseline measurements. Filled data points (black) indicate significance from vehicle measurements (*p* < 0.05). Data reflect mean ± SEM, n = 8.

Intraplantar administration of carrageenan produced significant (*p* < 0.0001) mechanical allodynia (Fig. 3) in vehicle treated mice compared to their pre-carrageenan baselines. Vehicle treatment did not alter mechanical allodynia thresholds throughout the time course tested, including 1 hour (*p* = 0.815), 2 hours (*p* = 0.271), and 3 hours (*p* = 0.271) following treatment.

RTIBW-16 (Fig. 3A) significantly attenuated mechanical allodynia at the 100 μg (*F* (2.36, 16.54) = 57.46, *p* < 0.001) and 56 μg (*F* (2.26, 15.83) = 42.61, *p* < 0.001) doses. Lower RTIBW-16 doses of 10 μg (*F* (2.93, 20.51) = 74.5, *p* < 0.001) and 5.6 μg (*F* (3.3, 23.12) = 35.31, *p* < 0.001) produced delayed-onset partial mechanical allodynia attenuation 3 hours after administration. A two-way ANOVA confirmed a significant interaction between RTIBW-16 treatment and time in the attenuation of mechanical allodynia in the carrageenan model (*F* (4.03, 28.18) = 9.23, *p* < 0.001).

NPB-23 (Fig. 3B) also attenuated mechanical allodynia in a dose- and time-dependent manner similar to RTIBW-16. The doses of 100 μg (*F* (3.62, 25.34) = 24.02, *p* < 0.001) and 56 μg (*F* (2.75, 19.26) = 27.76, *p* < 0.001) NPB-23 produced robust mechanical allodynia blockade in the carrageenan inflammatory pain model, Lower NPB-23 doses of 10 μg (*F* (2.01, 14.05) = 66.11, *p* < 0.001) and 5.6 μg (*F* (3.71, 25.95) = 107, *p* < 0.001) produced delayed-onset partial attenuation of mechanical allodynia 2 hours after administration. A two-way ANOVA confirmed a significant interaction between NPB-23 treatment and time in the attenuation of mechanical allodynia in the carrageenan model (*F* (5.30, 37.07) = 7.15, *p* < 0.001). At the 1 hour timepoint, the calculated ED_50_ values of RTIBW-16 and NPB-23 were 29.47 (21.71–40.00) and 19.92 (13.52–29.37) μg respectively (Table 1). In the carrageenan inflammatory pain model RTIBW-16 and NPB-23 were statistically equipotent in the reversal of mechanical allodynia.

Intraplantar carrageenan produced edema of the right hind paw as indicated by initial measurements at the 1 hour time point (Fig. 3C, D). Both RTIBW-16 (*F* (1.27, 8.86) = 5.47, *p* = 0.039) and NPB-23 (*F* (1.59, 11.16) = 241.6, *p* < 0.001) significantly reduced the magnitude of initial swelling as compared to vehicle treatment (Fig. 2C, D) although NPB-23 was more effective in reducing carrageenan-induced edema. A two-way ANOVA confirmed a significant interaction between RTIBW-16 treatment and time in the attenuation of paw edema in the carrageenan model (*F* (1.8, 12.61) = 10.51, *p* = 0.002). Likewise, a two-way ANOVA confirmed a significant interaction between NPB-23 treatment and time in the attenuation of paw edema in the carrageenan model (*F* (1.93, 13.52) = 42.65, *p* < 0.001).

## Discussion

Here we demonstrated that intrathecal administration of the truncated NPB-23 and its peptidomimetic RTIBW-16 displayed similar antinociceptive profiles as compared to naturally occurring ligands [19, 21, 22]. As all previous studies using NPB or NPW were conducted in rats [18, 20–23], the observation of antinociceptive effects of NPBWR1 agonists in mice in this study together with the hyperalgesic effect seen in NPB-deficient mice in response to inflammatory pain support the conserved role of NPBWR1 in pain processing across species [19].

Both RTIBW-16 and NPB-23 demonstrated antinociceptive effects in a panel of hot plate assay, CIPN, and carrageenan-induced inflammatory pain. They both produced maximum antinociceptive effect in the hot plate assay and exhibited equipotency in reversing mechanical allodynia in the CIPN and carrageenan-induced inflammatory pain models. However, they differed in onset, and duration of action. In the hot plate assay, RTIBW-16 exhibited a stronger antinociceptive effect at the earlier time point (0.5 hour), but with a shorter duration of action compared to NPB-23. In the CIPN assay, RTIBW-16 demonstrated a longer duration of action compared to NPB-23. While both RTIBW-16 and NPB-23 were statistically equipotent in the reversal of mechanical allodynia, NPB-23 was more effective in reducing carrageenan-induced edema. The variation in efficacy may stem from differences in the signaling pathways that drive the observed behaviors. Acute hot plate nociception occurs via nociceptive transmission and is mediated predominately via neuronal A*δ* and C fibers [40, 41]. CIPN mechanical allodynia arises from neurotoxicity and leads to peripheral nerve hyperexcitability, central sensitization, and enhanced synaptic efficacy at A*β* fibers [42, 43]. Although converging on similar downstream mechanisms as CIPN, carrageenan-induced mechanical allodynia arises from peripheral immune cell activation [1, 39]. The variations in their antinociceptive profiles are likely attributable to differences in their pharmacokinetic properties, which can influence factors such as onset and duration of action.

In conclusion, our studies demonstrate that NPBWR1 agonists exhibit robust antinociceptive activities across a range of pain models including acute thermal pain, inflammatory pain and neuropathic pain. The results underscore their potential as novel therapeutic agents for pain management. Notably, this is the first report of NPBWR1 agonists showing efficacy CIPN model, expanding the understanding of NBPWR1’s role in neuropathic pain. Future efforts should focus on the development of stable peptidomimetics to provide more potent and reliable tools for research and therapeutic use. These improved compounds would complement existing methods, such as naturally occurring compounds and genetic knockout or transgenic strategies, which are currently employed to explore the pharmacological profile of the NPBWR1. Additionally, mechanistic studies will be essential to elucidate the pathways underlying their analgesic effects, further advancing the field of pain research and drug development.

## Figures and Tables

**Table 1:** Calculated ED_50_ (μg) values for the effects of RTIBW-16 and NPB-23 as shown in Figs. 1 through 4 at 1 hour following treatment.

Experiment	Treatment	ED_50_ (μg)
**Hot Plate Latency**	RTIBW-16	42.86 (25.09–73.21)
	NPB-23	18.36 (10.17–33.15)
**Paclitaxel CIPN von Frey**	RTIBW-16	57.79 (53.13–95.06)
	NPB-23	45.06 (33.91–59.88)
**Carrageenan von Frey**	RTIBW-16	29.47 (21.71–40.00)
	NPB-23	19.92 (13.52–29.35)

Sample sizes are described in each figure legend. Values in parentheses are 95% confidence intervals.

## Data Availability

The data that support the findings of this study are openly available in the Open Science Framework repository at DOI 10.17605/OSF.IO/EP9FS.

## Abbreviations

% MPE: percentage maximum possible effect
ANOVA: analysis of variance
CIPN: chemotherapy–induced peripheral neuropathy
CNS: central nervous system
ED: effective dose
i.c.v.: intracerebroventricular
i.p.: intraperitoneal
i.t.: intrathecal
NPB: Neuropeptide B
NPW: Neuropeptide W
NPBWR: NPB Neuropeptide B/W Receptor
PE: polyethylene
